# Robotic rehabilitation for end-effector device and botulinum toxin in upper limb rehabilitation in chronic post-stroke patients: an integrated rehabilitative approach

**DOI:** 10.1007/s10072-021-05185-3

**Published:** 2021-04-07

**Authors:** Teresa Paolucci, Francesco Agostini, Massimiliano Mangone, Andrea Bernetti, Letizia Pezzi, Vitalma Liotti, Elena Recubini, Cristina Cantarella, Rosa Grazia Bellomo, Carlo D’Aurizio, Raoul Saggini

**Affiliations:** 1 Unit of Physical Medicine and Rehabilitation, Department of Oral Medical Science and Biotechnology (DSMOB), G. D’Annunzio University of Chieti-Pescara, Chieti, Italy; 2 Physical Medicine and Rehabilitation Unit, Department of Anatomical and Histological Sciences, Legal Medicine and Orthopedics, Sapienza University of Rome, Piazzale Aldo Moro 5, 00185 Rome, Italy; 3 U.O.C. Physical Medicine and Rehabilitation, Hospital of Popoli, Pescara, Italy; 4 Department of Biomolecular Sciences, University of Study of Urbino Carlo Bo, Urbino, Italy; 5 IRCSS Centro Neurolesi “Bonino Pulejo”, Messina, Italy

**Keywords:** Evaluation model, Motion control skill, Robotic end-effector kinematic feature, Surgical robot system

## Abstract

**Background:**

Determine the effects of an integrated rehabilitation protocol, including botulinum toxin and conventional rehabilitation exercise plus end-effector (EE) robotic training for functional recovery of the upper limb (UL) compared to training with the robot alone in post-chronic stroke patients with mild to severe spasticity, compared to training with the robot alone.

**Methods:**

In this prospective, observational case-control study, stroke patients were allocated into 2 groups: robot group (RG, patients who underwent robotic treatment with EE) and robot-toxin group (RTG, patients who in addition have carried out the injection of botulinum toxin for UL recovery). All patients were assessed by Fugl-Meyer Assessment (FMA), Motricity Index (MI), modified Ashworth scale (MAS), numeric rating scale (NRS), Box and Block Test (BBT), Frenchay Arm Test (FAT), and Barthel Index (BI) at baseline (T0), T1 (end of treatment), and T2 (3 months of follow-up).

**Results:**

Forty-four patients were included and analyzed (21RG; 23RTG). From the analysis between groups, the results suggested how there was a statistically significant difference in favor of RTG, specifically ΔT0-T1 and ΔT0-T2 for B&B *p* = 0.009 and *p* = 0.035; ΔT0-T1 and ΔT0-T2 for FAT with *p* = 0.016 and *p* = 0.031; ΔT0-T1 for MAS shoulder *p* = 0.016; ΔT0-T1 and ΔT0-T2 with *p* = 0.010 and *p* = 0.005 for MAS elbow; and ΔT0-T1 and ΔT0-T2 with *p* = 0.001 and *p* = 0.013 for MAS wrist.

**Conclusion:**

Our results suggest, in line with the literature, a good efficacy in the reduction of spasticity and in the improvement of the function of the UL, with the reduction of pain, adopting a rehabilitation protocol integrated with BoTN, robot-assisted training, and traditional physiotherapy.

## Introduction

Rehabilitation represents a very important focus for motor recovery after stroke which influences the neurobiology of neuronal plasticity providing controlled, repetitive, and variable patterns. After a stroke, approximately 80% of patients report a UL motor deficit with all subsequent limitations in activities of daily living and limiting social participation [1, 2].

For the functional recovery of the upper limb (UL), training with specific repetitive task exercises with progressive difficulty, goal oriented, is recommended. In addition, task-oriented exercises in the recovery of UL function should be tailored and personalized as much as possible considering the patient’s possibilities and needs [3, 4]. From these premises, robotic devices for UL open a window to define therapeutic modalities as a possible beneficial drug, in patients with a moderate-to-severe deficit after stroke, able to boost biological, neurobiological, and epigenetic changes in the central nervous system (CNS) [5]. We can consider three main functional consequences of impairments on UL function after stroke: (1) learned nonuse, (2) learned bad use, and (3) forgetting use [6], where robotic rehabilitation with both the end-effector system (EE) and the exoskeleton (ES) can be effective in the recovery alone or in association with conventional therapy. For example, during rhythmic pointing movements of the UL and the hand, end-effector and joint angles are reciprocally related in synergies and the action system is organized as a complex dynamical system [7–9]; then, robotic training exercises could improve the fine and purposeful motor organization of the UL as in acute as in chronic phase after stroke. Moreover, recovery of the UL function recognizes a recovery time of over 1 year that is likely mediated by a complex combination of spontaneous and learning-dependent processes, including restitution, substitution, and compensation. In particular, upper extremity impairments have chronic effects on functional independence and satisfaction in 50 to 70% of all stroke patients [10, 11]. Therefore, the rehabilitation of UL in the post-stroke patient represents a very important aspect to integrate with conventional therapy. From this point of view, robotic therapy represents a rehabilitation resource that has given very encouraging results in recent years. The UL robotic-assisted therapy combined with conventional therapy during the early rehabilitation phase after stroke is more effective than conventional therapy alone to improve gross manual dexterity, upper limb ability during functional tasks, and patient social participation [12, 13]. Also, Stephanie Hyeyoung Lee and colleagues studied that the EE robot intervention is better than the Exo-robot intervention with regard to activity and participation among chronic stroke patients with moderate-to-severe UL impairment [14]. Very interesting is the study by Mazzoleni et al., which pointed out how a haptic device in chronic stroke patients reported significant changes on the elbow spasticity [15]. Also, other authors demonstrated improvement in motor function and in muscular activation pattern after a short robotic training in chronic post-stroke spasticity of UL treated prior to the treatment with botulinum toxin; instead, in less severe spasticity, the only robotic treatment could be effective [16]. Deepening the topic, Gandolfi et al. showed that the combined use of robot-assisted UL training and botulinum toxin (BoNT) appears to be a promising therapeutic synergism to improve UL function in chronic stroke patients, but only the robot-assisted UL training contributed to improving muscle strength [17]. The robotic rehabilitation should have to include task-specific and context-specific training as principles in motor learning, and the training should target the goals that are relevant for the needs of patients.

In the context of pharmacological treatment, in association with the rehabilitation plan, the use of botulinum toxin (BT) is indicated for the treatment of focal spasticity in the hemiplegic patient, especially for the recovery of the UL [18]: BT type A improved muscle tone, physician global assessment, and disability assessment scale in upper limb spasticity and increases the Fugl-Meyer score in lower limb spasticity [19]. Also, Andringa et al. in a recent systematic review concluded that no further trials are needed to investigate BoNT for its favorable effects on resistance to passive movement of the spastic wrist and fingers, and on self-care [20]. In light of these premises and the indications of the literature regarding UL robotic rehabilitation in the post-stroke patient, the aim of our research was to determine the effects of an integrated rehabilitation protocol, including botulinum toxin and conventional rehabilitation exercise with EE robotic training for functional recovery of UL in post-chronic stroke patients with mild to severe spasticity, compared to training with the robot alone. The primary outcome was the assessment of spasticity and the secondary outcome was the recovery of UL function.

## Materials and methods

Patients with chronic stroke (> 6 months) (time from stroke 12–24 months) of both sexes who were attending the outpatient rehabilitative unit of SS Trinità Hospital of Popoli (Italy) from September 2018 to December 2019 were enrolled [21].

The inclusion criteria were previous stroke (ischemic/hemorrhagic) documented at neuroradiological examinations (CT/MRI) with upper limb functional deficits (associated or not with leg paresis), with a score > 2 at MRC test, with a sensory-motor deficit, monoparesis, hemiparesis; age > 18 years; Mini-Mental Examination Score > 24 [22–24], BMI < 30; possibility of maintaining the sitting position. Patients were excluded if they presented with any of the following: bilateral impairment; neglect according to barrage test and clinical judgment; cognitive or behavioral impairment such as to affect the understanding or execution of robotic training; inability or unwillingness to give informed consent; severe comorbidities.

The research was approved by the Local Hospital Committee of Popoli and was approved by the Department of Oral and Biotechnological Medical Sciences of the G. D’Annunzio University of Chieti (Italy) (315/20).

The study complies with the principles of the Helsinki Declaration in accordance with good clinical practice. All patients gave written informed consent after receiving detailed information on the study’s aims and procedures. Patient personal data are processed anonymously with an alphanumeric code and electronically stored.

Clinical data were collected at T0 (baseline), T1 (at the end of the treatment), and T2 (after 3 months of follow-up). Adverse events were also registered during the follow-up.

### Study design

The prospective, observational case-control study was conducted following the Strobe Guidelines [25].

Stroke patients were consecutively enrolled and allocated into 2 groups by 1:1 sample randomization: robot group (RG—patients who underwent robotic treatment with end-effector and conventional treatment) and robot-toxin group (RTG—patients who in addition to robotic treatment and conventional treatment) have carried out the injection of botulinum toxin for upper limb recovery). To reduce bias, the physician who administered the rating scales was blinded to the patient’s group allocation, and the researcher who analyzed the data was blinded to the therapy.

All patients in both groups underwent conventional rehabilitation treatment for UL.

### BoTN treatment—target muscles and drug dosage

RTG patients underwent BoNT-A injection in the paretic upper limb. The inoculations were always carried out after clinical and functional evaluation, in order to plan the muscles to be infiltrated and the dose for each muscle also in relation to the patient’s response to previous inoculations. In fact, the same administration scheme was not always followed; an example is given by the flexor of the first finger, whose re-inoculation was often not necessary. The infiltrations were always performed, after palpation of the muscle bellies, with ultrasound guidance (MYlabfive, ESAOTE) in order to minimize the risk of errors and avoid inoculation into the vessels. The drug dilution was done with 2CC of saline, and 2 points were infiltrated for each muscle.

We chose to treat the following muscles as necessary: brachial biceps (BB), brachioradialis (BR), pronator teres (PT), flexor carpi radialis (FCR), flexor carpi ulnaris (FCU), flexor digitorum superficialis (FDS), flexor digitorum profundus (FDP), and flexor digitorum longus (FDL). Two commercial formulations of BoNT were used according to hospital availability (Botox®-Allergan Inc; Dysport®-Ipsen Biopharm Ltd), and the dose injected for each muscle was based on the severity of spasticity, up to the maximum dose approved for the treatment of spasticity of the upper limb in adults. The number of injection sites per muscle and the dose injected into each muscle were determined at the discretion of the investigator, physician in physical medicine, and rehabilitation, according to the USPRM spasticity approach [26] (Table 1).

**Table 1:** Muscles treated and dosages

Muscle	Dysport (IU)	Botox (IU)
Biceps brachial head medial	100	40
Biceps brachialis head lateral	100	40
Subscapularis (in a few cases)	200	-
Brachialis	150–200	60–70
Brachioradialis	150–200	60–70
Round pronator	200–250	70–80
Radial flexor of the carpus	150–200	60–70
Ulnar flexor of the carpus	150	60
1st finger flexor	150	60
Superficial flexor of the fingers	150–200	60–70
Deep flexor of the fingers	150–200	60–70

*IU*, international unit

### Rehabilitative treatment

#### Robotic end effector

**Fig. 1:**
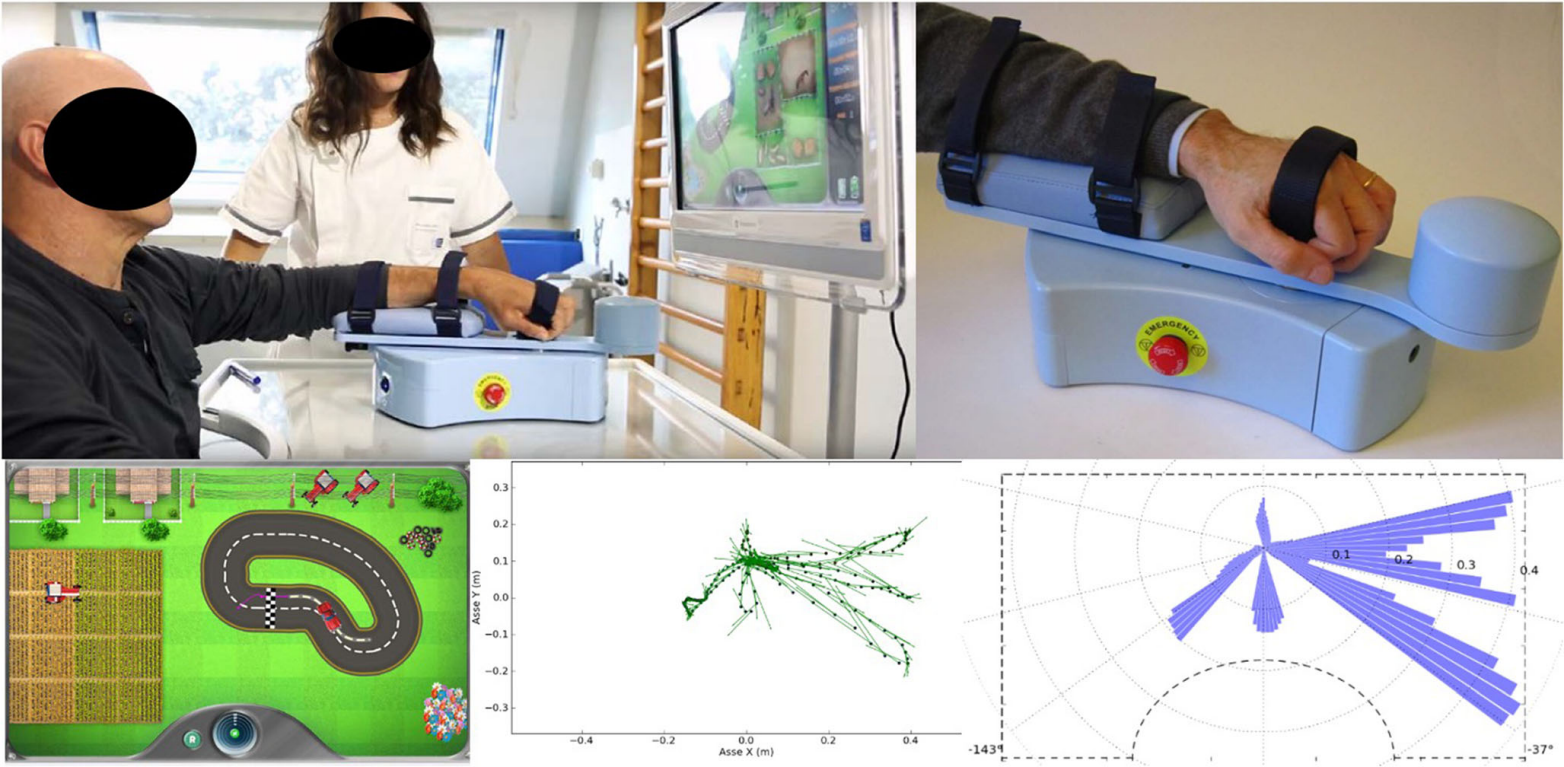
MObile roboT for upper limb neuroOrtho Rehabilitation (MOTORE)

**Fig. 2:**
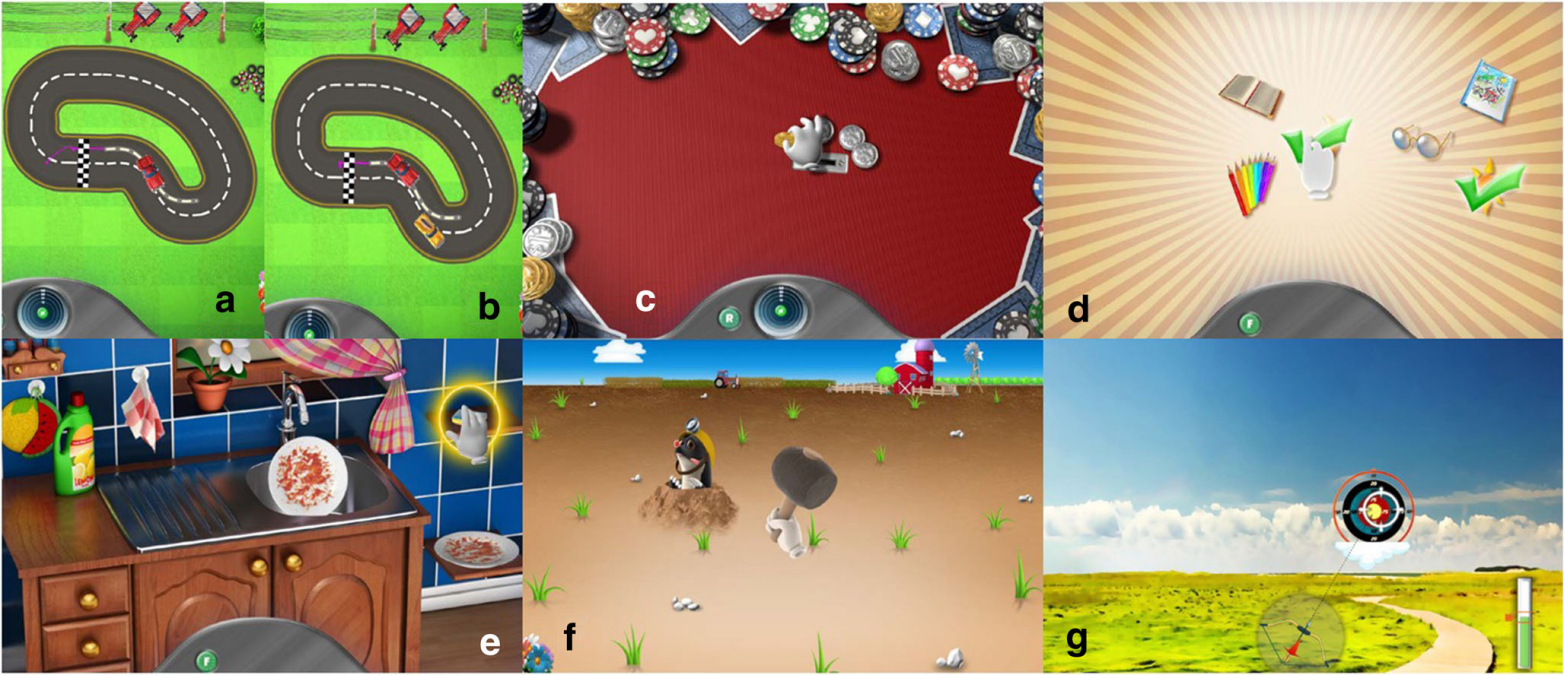
Robot end-effector protocol. **a** Trajectories; **b** pursuit; **c** coins; **d** memory; **e** washing dishes; **f** moles; **g** archery

All outpatients recruited underwent robotic treatment with “MOTORE - *MObile roboT for upper limb neuroOrtho REhabilitation*” (Humanware, Italy), which is a device for upper limb rehabilitation, offering different modes of operation, ranging from guided support for highly functional patients to assisted or unassisted movement for patients with severe hemiplegia that supports, helps, and/or opposes the movement according to the rehabilitation goals. The device allows patients to perform exercises with visual feedback that let the execution of planar (bidimensional) movements, like reaching and carrying exercises with the intention to rehabilitate the shoulder and elbow areas. The graphical interface allows exercises for carrying out predetermined trajectories (circular or oval) to improve ROM and relearn how to dose strength, exercises that simulate daily life activities (washing dishes, taking coins) and exercises that train cognitive functions (memory, logical deductive functions) (Fig. 1). Considering also the protocol of Aprile et al. [27, 28], the patients performed every evaluation task three times and repeated every reaching movements from 8 to 12 repetitions as follows: (A) trajectories (the patient is asked to drive one of the selectable tracks with his car); (B) pursuit (the patient is asked to chase the opposing car with his car along one of the selectable tracks); (C) coins (the patient is asked to grab some coins and bring them back towards the center of the work surface, where they will accumulate. For this exercise, the patient will perform “fan” movements); (D) memory (the patient is asked to make associations between pairs of images being free to move on the plane without having to follow a particular trajectory); (E) washing dishes (the patient is asked to wash the dishes according to a predetermined sequence of actions); (F) moles (the patient is asked to crush the moles that come out of the ground; (G) archery (the patient is asked to exert an appropriate force on the handpiece of the manipulandum in the opposite direction to that joining the arch and the center of the target, as when loading the bow before firing the arrow) (Fig. 2). The treatment was performed daily for 30 min, 3 days a week, for a total of 20 sessions. Robotic therapy was started seven days after botulinum toxin infiltration.

#### Conventional rehabilitative treatment

In addition, after the 30 min of robotic training, patients in both groups performed passive mobilization exercises on the main joints of the UL and stretching (shoulder, elbow, wrist, and fingers), scapular joint mobilization exercises, and cervical spine “pompage” exercises: the rehabilitation maneuvers were performed by the physiotherapist, expert in neurological rehabilitation, on the couch with the patient in a prone or supine position as needed. The protocol also included active movements of the UL against the force of gravity, with the final request of isometric holding of the position for at least 6 s. According to the patient’s resources, the physiotherapist, during the treatment, increased the joint range during passive mobilization and the number of repetitions, starting from a minimum of 5 repetitions up to 15 repetitions at least twice, with a rest break from 2 to 3 min. The stretching exercises were performed statically for at least three times during the session. Conventional treatment followed robotic treatment for organizational needs of the rehabilitation department and had the same duration as robotic therapy, to also facilitate the patient as it was an outpatient rehabilitation therapy, 3 days a week, for a total of 20 sessions

### Outcome measure

#### Fugl-Meyer Assessment scale (FMA)

The FM scale is a 226-point multi-item Likert-type scale that was developed as a stroke-specific, performance-based impairment index. It is designed to assess motor functioning, balance, sensation, and joint functioning in patients with post-stroke hemiplegia. Each domain contains multiple items, each of which is scored on a 3-point ordinal scale (0 = cannot perform, 1 = performs partially, 2 = performs fully). The FMA-UL motor domain is most widely used and has the primary value of monitoring motor recovery after stroke specifically for the upper limb (maximum score 66 points) [29].

#### Motricity Index (MI)

The muscle weakness of the paretic upper extremity was quantified using the Motricity Index. It involves grading strength based on a patient’s ability to activate a muscle group, to move a limb segment through a range of motion, and to resist the force of an examiner. The scale includes three actions: pinch grasp, elbow flexion, and shoulder abduction, and these are each scored (0–33) with a maximum possible score of 100 (adding one to the sum of the three actions) for the upper limb [30].

#### Modified Ashworth scale (MAS)

The modified Ashworth scale is the most widely clinical scale used to measure muscle spasticity. It is used to assess the resistance experienced during the passive range of upper limb motion (shoulder adductors, elbow, and wrist flexors), which does not require any instrumentation and is quick to perform. The scale consists of a 5-point nominal scale using subjective clinical assessments of tone ranging from 0—“no increases in tone”—to 4—“limb rigid in flexion or extension (abduction/adduction).” An additional grade is added (1+) for the MAS to indicate resistance in the movement [31].

#### Numeric rating scale (NRS)

Numerical rating scale (NRS) is the simplest and most commonly used scale to assess pain; the range is from 0 to 10, with 0 being “no pain” and 10 being “the worst pain imaginable” [32].

#### Box and Block Test (BBT)

The BBT is a quick, simple, and reliable measurement, and it is used to assess the unilateral gross manual dexterity. The BBT consists of moving the maximum number of blocks from one compartment of a box to another, one by one, within 1 min [33].

#### Frenchay Arm Test (FAT)

The FAT assesses upper extremity specific measure of activity limitation. It is an upper extremity proximal motor control and dexterity during ADL performance in patients with impairments of the upper extremity resulting from neurological conditions [34].

#### Barthel Index (BI)

The BI was introduced by Mahoney and Barthel (1965) and later modified by Collin et al. (1988) and Shah et al. (1989). The original 10-item form of the BI comprises 10 common activities of daily living (ADL): “feeding,” “bathing,” “grooming,” “dressing,” “bowel” and “bladder control,” “toilet use,” “transfers (bed to chair and back),” “mobility,” and “stair climbing.” The items are rated as whether patients can perform the activities independently or with assistance or are totally dependent (scored 10, 5, or 0 respectively, or from 15 to 0 for transfers and mobility) [35–38].

### Sample size calculation

The G*Power version 3.1.9.2 program was used to evaluate the required sample size.

We used the difference in time (ΔT) of both the Fugl-Meyer Assessment (FMA) and the Motricity Index (MI) values as an outcome for the calculation of the sample size, inserting the following values for the FMA scale mean = 5.29, SD = 5.02 and for the MI mean = − 0.67, SD = 0.73 [16]; a significance level of 0.05 and a power level of 0.95; the sample size required is respectively 13 and 15 per group.

### Statistical analysis

Values are expressed as median and minimum and maximum for continuous variables and proportion for categorical variables, as appropriate. Demographic and clinical data at baseline included the following parameters: age and BMI (body mass index) expressed in median and minimum and maximum, gender (female or male), hemiplegic side (left or right), and dominant side (left or right). Differences in baseline characteristics between the 2 treatment groups were analyzed by Fisher’s exact test or Mann-Whitney *U* test, as appropriate. The analysis of time difference in the two groups (robot group, RG and robot + toxin group, RTG) was performed through a Friedman analysis for repeated measures to determine if there were differences in the different evaluation times in the 2 groups. A subsequent analysis for each parameter, a pairwise comparison, was performed with a Bonferroni correction. The Mann-Whitney *U* test was used for all the parameters studied to evaluate the time differences between the groups and the variations (Δ) between T0 and T1, T1 and T2, and T2 and T0. All primary and secondary outcomes analyzed were performed according to the principle of intention-to-treat analysis. A value of *p* < 0.05 was considered significant. Statistical analyses were carried out using the SPSS version 18 package (SPSS Inc, Chicago, IL, USA).

## Result

**Fig. 3:**
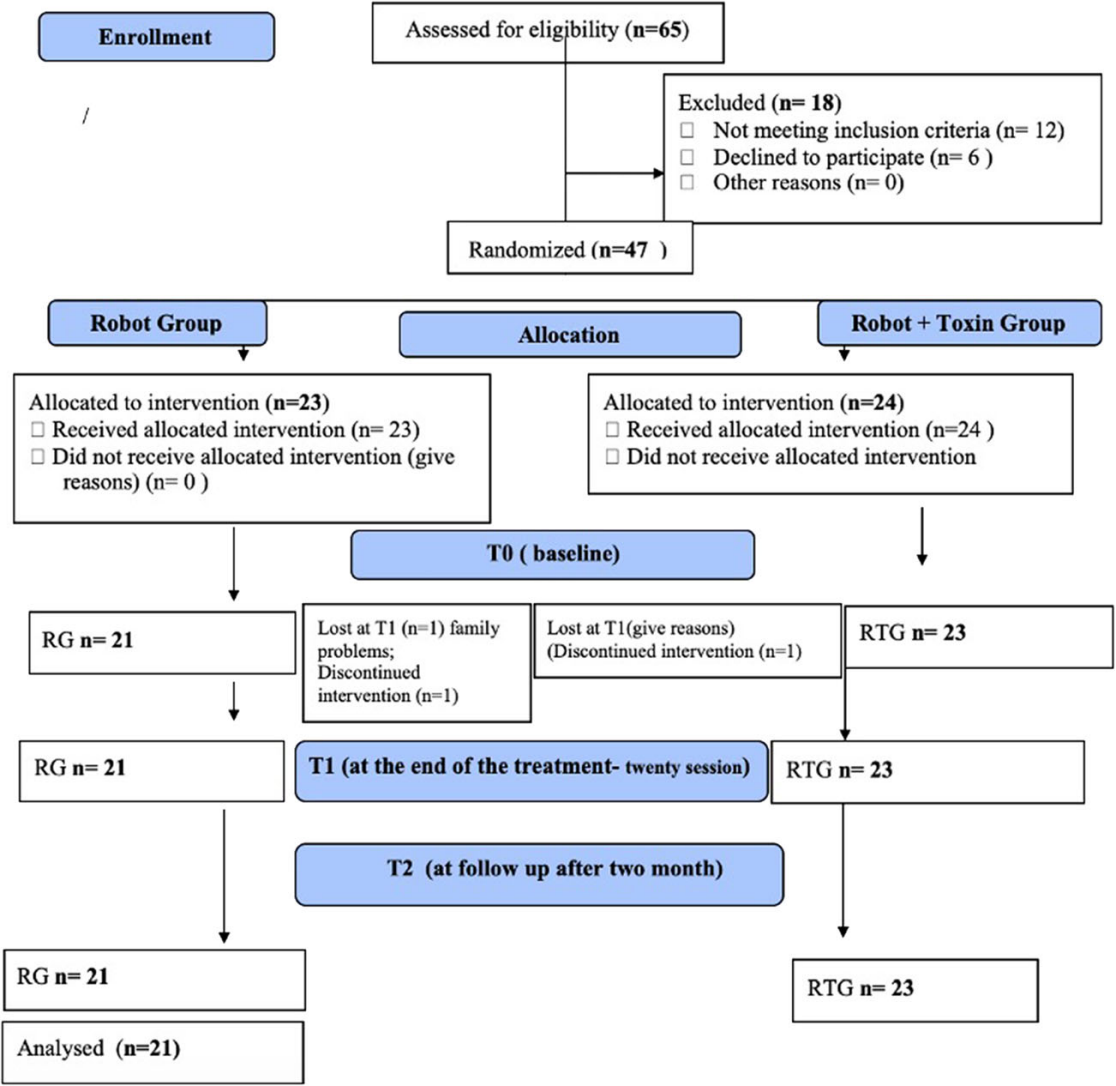
Flowchart

Sixty-five (*N* = 65) patients were observed and forty-seven were enrolled respect the inclusion criteria. Specifically, data of forty-four (*N* = 44) patients were included and analyzed. We observed a dropout of 2 patients in the RG group of which 1 due to discontinuity to treatment and 1 for family reasons; on the other hand, in the RTG, we observed 1 dropout due to discontinuity in treatment. Then, the data of 21 patients in the RG and 23 patients in the RG were analyzed (Fig. 3).

Two patients in RG and one patient in RTG, respectively, did not complete the rehabilitative program: one patient in the RG was excluded because he did not constantly participate in the rehabilitation program and one for a personal problem, instead, one patient was excluded in the RTG because he did not perform all the rehabilitative sessions. No adverse events were reported during rehabilitative treatment and toxin treatment.

A descriptive analysis was performed as showed in Table 2 for age, body mass index (BMI), male/female), hemiplegic side (left or right), and dominant side (left or right). Compared to the baseline, the two groups are homogeneous and match for age with a mean age (years) of 65 ± 10.09 (RG, males 67%) and 65.7 ± 10.5 (RTG, males 43.5%) for *P* = 0.487, BMI 26.8 ± 2.3 (RG) and 26.9 ± 1.8 (RTG) for *P* = 0.567.

**Table 2:** Clinical parameters

Clinical parameters	Robot group (21)	Robot + toxin group (23)	*P* value
Age (years–mean standard deviation))	65 ± 10.09	65.7 ± 10.5	0.487
BMI (mean standard deviation)	26.8 ± 2.3	26.9 ± 1.8	0.567
Gender (%)	67 M; 33 F	43.5 M; 56.5 F	0.122
Hemiplegic side (%)	57 left; 43 right	56.5 left; 43.5 right	0.309
Dominant side (%)	95 right; 5 left	100 right; 0 left	-
Type of stroke (%)	14.3% TACS*; 66.7% PACS*; 19.0 hemorrhagic stroke	30.4% TACS*; 56.5% PACS*; 13.1 hemorrhagic stroke	0.724
FMA (median, range)	37 (16–58)	34 (23–54)	0.759
MAS shoulder (median, range)	1 (1–4)	1 (1–4)	0.159
MAS elbow (median, range)	1 (1–4)	2 (1–4)	0.163
MAS wrist (median, range)	1 (1–4)	2 (1–4)	0.184
Time from stroke (months–mean standard deviation)	8.5 ± 2.6		

*BMI*, body mass index; mean and standard deviation; *TACS*, total anterior circulation stroke; *PACS*, partial anterior circulation stroke

*Bamford classification

From the analysis *between groups*, the results suggested how there was a statistically significant difference for *p* < 0.05 in favor of the RTG versus RG group as reported in Table 3, specifically Δ T0-T1 and Δ T0-T2 for B&B *p* = 0.009 and *p* = 0.035; Δ T0-T1 and Δ T0-T2 for Frenchay Arm Scale with *p* = 0.016 and *p* = 0.031; Δ T0-T1 for MAS shoulder *p* = 0.016; Δ T0-T1 and Δ T0-T2 with *p* = 0.010 and *p* = 0.005 for MAS elbow; and Δ T0-T1 and Δ T0-T2 with *p* = 0.001 and *p* = 0.013 for MAS wrist.

**Table 3:** Statistical analysis between groups

	Robot group	Robot + toxin group	*p* value
ΔT0-T1 FMA	10 (− 1–28)	7 (1–27)	0.689
ΔT1-T2 FMA	2 (0–20)	4 (− 2–13)	0.558
ΔT0-T2 FMA	12 (2–30)	15 (3–38)	0.972
ΔT0-T1 MI UL	16 (0–50)	15 (3–41)	0.317
ΔT1-T2 MI UL	0 (0–24)	2 (0–10)	0.210
ΔT0-T2 MI UL	18 (0–50)	20 (7–43)	0.503
ΔT0-T1 B&B	5 (− 11–49)	12 (4–31)	0.009
ΔT1-T2 B&B	0 (− 2–22)	2 (0–12)	0.077
ΔT0-T2 B&B	9 (0–49)	16 (7–36)	0.035
ΔT0-T1 Frenchay Arm	1 (0–5)	2 (0–4)	0.016
ΔT1-T2 Frenchay Arm	0 (0–2)	0 (0–1)	0.249
ΔT0-T2 Frenchay Arm	1 (0–5)	2 (0–5)	0.031
ΔT0-T1 MAS shoulder	0 (− 3–1)	− 1 (− 3–1)	0.016
ΔT1-T2 MAS shoulder	0 (− 1–2)	0 (− 1–1)	0.576
ΔT0-T2 MAS shoulder	0 (− 3–2)	− 1 (− 3–1)	0.054
ΔT0-T1 MAS elbow	0 (− 2–2)	− 1 (− 3–0)	0.010
ΔT1-T2 MAS elbow	0 (− 3–2)	0 (− 1–0)	0.282
ΔT0-T2 MAS elbow	0 (− 2–1)	− 1 (− 4–0)	0.005
ΔT0-T1 MAS wrist	0 (− 4–2)	− 1 (− 4–1)	0.001
ΔT1-T2 MAS wrist	0 (− 2–2)	0 (− 1–0)	0.176
ΔT0-T2 MAS wrist	0 (− 4–2)	− 1 (− 4–0)	0.013
ΔT0-T1 NRS	0 (− 6–5)	− 2 (− 8–1)	0.258
ΔT1-T2 NRS	0 (− 3–2)	0 (− 1–0)	0.366
ΔT0-T2 NRS	0 (− 6–2)	− 2 (− 8–1)	0.188
ΔT0-T1 BI	10 (0–65)	16 (0–53)	0.715
ΔT1-T2 BI	0 (− 15–45)	2 (0–10)	0.668
ΔT0-T2 BI	25 (− 10–80)	19 (4–57)	0.525

Legends: Δ = difference; *FMA*, Fugl-Meyer Assessment scale; *MI*, Motricity Index; *MAS*, modified Ashworth scale; *NRS*, numeric rating scale; *BBT*, Box and Block Test; *FAT*, Frenchay Arm Test; *BI*, Barthel Index; baseline (T0), T1 (at the end of the treatment), and T2 (after 3 months of follow-up)

From the analysis *within group*, the results showed an improvement over time compared for all parameters in the RTG; instead, in the RG, the results underlined a constant trend for MAS and NRS without a statistically significant difference over time (Table 4).

**Table 4:** Statistical analysis within group

	Group	Evaluation times				Post hoc		
		T0	T1	T2	*p* value	*p* T0-T1	*p* T1-T2	*p* T0-T2
FMA	Robot group	37 (16–58)	46 (20–65)	55 (20–65)	< 0.001	0.001	0.160	< 0.001
MI UL	Robot group	50 (28–76)	66 (28–100)	66 (28–100)	< 0.001	0.002	0.370	< 0.001
B&B	Robot group	11 (0–34)	23 (0–53)	27 (0–53)	< 0.001	0.005	0.570	< 0.001
Frenchay arm	Robot group	2 (0–5)	4 (0–7)	4 (0–7)	< 0.001	0.050	0.950	0.002
MAS shoulder	Robot group	1 (1–4)	1 (0–2)	0 (0–3)	0.900	-	-	-
MAS elbow	Robot group	1 (1–4)	1 (0–3)	1 (0–3)	0.050	-	-	-
MAS wrist	Robot group	1 (1–4)	1 (0–4)	0 (0–4)	0.060	-	-	-
NRS	Robot group	1 (0–10)	0 (0–10)	0 (0–10)	0.060	-	-	-
BI	Robot group	65 (20–90)	80 (30–100)	90 (55–100)	< 0.001	0.002	1	< 0.001
FMA	Robot + toxin group	34 (23–54)	49 (31–65)	52 (34–72)	< 0.001	< 0.001	0.060	< 0.001
MI UL	Robot + toxin group	50 (28–72)	67 (42–100)	68 (44–100)	< 0.001	0.001	0.001	< 0.001
B&B	Robot + toxin group	4 (0–33)	23 (6–53)	26 (12–58)	< 0.001	0.002	0.004	< 0.001
Frenchay arm	Robot + toxin group	2 (0–5)	3 (2–5)	4 (3–5)	< 0.001	0.001	0.314	< 0.001
MAS shoulder	Robot + toxin group	1 (1–4)	0 (0–1)	0 (0–1)	< 0.001	0.012	1	0.045
MAS elbow	Robot + toxin group	2 (1–4)	1 (0–2)	0 (0–1)	< 0.001	0.015	1	0.001
MAS wrist	Robot + toxin group	2 (1–4)	0 (0–2)	0 (0–1)	< 0.001	< 0.001	1	< 0.001
NRS	Robot + toxin group	2 (0–10)	1 (0–4)	1 (0–3)	0.001	0.081	1	0.012
BI	Robot + toxin group	67 (25–92)	82 (58–100)	82 (60–100)	0.007	**-**	-	-

Legends: *FMA*, Fugl-Meyer Assessment scale; *MI*, Motricity Index; *MAS*, modified Ashworth scale; *NRS*, numeric rating scale; *BBT*, Box and Block Test; *FAT*, Frenchay Arm Test; *BI*, Barthel Index (BI); baseline (T0), T1 (at the end of the treatment), and T2 (after 3 months of follow-up)

## Discussion

Considering the scope of our research, the results were encouraging. The data reported confirm the validity of robotic-assisted therapy for the rehabilitation of the UL in the chronic post-stroke patient: both groups report a statistically significant improvement after treatment with the EE system for the FMA scale. The enrolled patients also demonstrated good compliance to the rehabilitation treatment (dropout < 20%), reporting no adverse effects in the group subjected to infiltration with BoTN. Also, no side effects have been described also for robotic rehabilitation as well as for infiltration with BoTN.

The EE system allowed the integration of cognitive stimuli with motor stimuli through feedback stimulus (as visual and auditory cues), biofeedback, VR, and exergame augmented feedback during training exercises. Thus, during exercise robot training, the bottom-up motor rehabilitation component (motor intensity and task oriented) and the top-down (attention, visuo-spatial ability) motor cognitive rehabilitation component are integrated with robots [39]. Robotic treatment should be considered a rehabilitation tool useful to generate a more complex, controlled multisensory stimulation of the patient and useful to modify the plasticity of neural connections through the experience of movement [6].

Our results, underline how, in the experimental group (RTG) where the infiltration with BoTN was carried out before robot training, parallel to the reduction of spasticity, a statistically significant difference was observed between the two groups not only for the MAS (shoulder, elbow, and wrist) at T1 and for the MAS wrist also at T2 but also compared to manual dexterity for the B&B scale at T1 and T2 and the Frenchay Arm Scale, always at both T1 and T2.

Furthermore, the treatment with BoTN would have a different action also in the remodeling of the plasticity of the recovery of the UL movement, in patients with chronic stroke [40]: different effects of BoNT lowered spasticity on sensorimotor networks for mild or severe weakness in hand weakness.

Considering the study by Gandolfi et al., the results suggested an increase in muscle strength on UL in the robot-assisted UL group, an improvement in shoulder range of motion in particular abduction and external rotation, and an improvement in elbow flexion. In this study, having both the experimental and control groups, treated with BoNT, no differences in the MAS and FMA scales were reported. Compared to our protocol, the authors proposed a robot-assisted treatment for UL of two sessions/week lasting 45 min for 10 sessions in total, while our protocol envisaged three sessions per week for a total of 20 sessions. In addition, in our integrated rehabilitative approach, after the 30-min robotic session, patients in both groups performed passive mobilization exercises on the main joints of the UL and stretching [17].

We cannot forget that in chronic stroke patients, spasticity and the reduction of UL function represent one of the main interventions of the rehabilitation requests. Furthermore, in the first year after a stroke, up to 38% of patients may report spasticity of the UL (range from 7 to 38%), and the precocity of the targeted rehabilitation intervention can avoid the onset of viscoelastic changes in the muscle and soft tissue, which would affect the joint recovery of the UL [41–44].

The robot training in the recovery of UL respects the characteristics of being task specific, repetitive, and motivating for the patients, who often have depressive disorders and poor alliance to rehabilitation treatment [45–47].

Other authors, such as Pennati et al., adopt a rehabilitation protocol of 10 sessions with robot training lasting 60 min, two or three times a week: the data showed that in some chronic stroke patients with focal UL spasticity, botulinum toxin does not add further improvements if performed together with robotic training. These results, although very interesting, must be interpreted in the light of the study design as a pilot with a small sample size [16]. However, the starting hypothesis is supported by the literature and embraces both the importance of how the treatment of UL spasticity should always consider both the neurogenic component and the peripheral component of spasticity and of how robotic training does not increase hypertonia but reduce the spasticity of the antagonist muscles, through a mechanism of reciprocal inhibition [48–51].

In a recent multi-center study by Aprile et al., robot training for UL was performed in the rehabilitation of stroke patients with a daily frequency (5 days a week) for 45 min, for a total of 30 sessions. The authors reported that there are no statistically significant differences in functional recovery of UL in patients with subacute stroke, the outcomes being equivalent with respect to conventional rehabilitative therapy. Instead, it was highlighted that, in the group of patients who carried out the robotic training, the subjects reported an increase in strength on the UL, probably related to the high number of repetitions and to a more intense training [27]. Also, in patients with long-term upper limb deficits after stroke, other authors suggested a similar trend with a better result for 12-week robot-assisted therapy group with respect to usual care for FM score but the differences were not significant [52].

From the analysis *within group*, the results showed an improvement over time compared for all parameters in the RTG; instead, in the RG, the results underlined a constant trend for MAS and NRS without a statistically significant difference over time (Table 4). Yelnik et al. [53], in their randomized, double-blind, placebo-controlled, two-parallel-group study investigated the beneficial effect of injection of botulinum toxin A (single-dose botulinum toxin—500 Speywood units) into the muscle subscapularis for shoulder pain in stroke patients with spastic hemiplegia. Pain was assessed using a 10-point verbal scale. Upper limb spasticity was assessed using the MAS for the medial rotators of the shoulder and for the flexors of the elbow, wrist, and fingers. Assessments were performed at baseline and at weeks 1, 2, and 4. A clinically significant improvement in the passive lateral rotation was observed, resulting from a decrease in local spasticity. External rotation is greatly improved, more so than abduction, which is not surprising because the subscapularis muscle is a strong internal rotator with little impact on abduction. In conjunction with the improvement in shoulder pain and mobility, spasticity of the upper limb muscles appeared to be reduced. The authors conclude that subscapular injection of botulinum toxin A appears to be useful in the management of shoulder pain in spastic hemiplegic patients. The reduction of pain by subscapular injection of botulinum toxin A, with a concomitant improvement in the range of motion of the shoulder observed in the present study, therefore, in line with our results, confirms the role of spasticity in post-stroke shoulder pain.

### Limits

The positive effect of this therapy with respect to neuroplasticity is hypothesized but not studied in this paper. The only clinical analysis (or with a screening tool such as the Mini Mental) does not allow a more reliable verification of the percentage of subjects who cannot access this treatment due to neuropsychological limitations. In this study, muscle recruitment with electromyography (EMG) was not assessed. The absence of instrumental evaluation (for example h-reflex, dynamometer, gait analysis, or correlation between the variation of spasticity and muscle strength) of the observed phenomena certainly represents a limitation. The clinical judgment alone was used to investigate the presence of neglect. The assessments at T0, T1, and T2 and the related statistical results between and within the groups, using the “MOTORE” test and related data (force, acceleration, speed), were not included in the manuscript because we focused on the functional recovery of the patients with chronic stroke. The lack of analysis of the level of aphasia represents a limitation.

## Conclusion

The spasticity of UL together with functional reduction and painful, in chronic stroke patients, is the primary reason for the request for rehabilitation intervention. Our results suggest, in line with the literature, a good efficacy in the reduction of spasticity and in the improvement of the function of the UL, with the reduction of UL pain, adopting a rehabilitation protocol integrated with BoTN, robot-assisted training, and traditional physiotherapy.
